# Molecular Characterization of Antimicrobial Resistance and Virulence Genes of Bacterial Pathogens from Bovine and Caprine Mastitis in Northern Lebanon

**DOI:** 10.3390/microorganisms9061148

**Published:** 2021-05-27

**Authors:** Zahie Abboud, Lucia Galuppo, Marco Tolone, Maria Vitale, Roberto Puleio, Marwan Osman, Guido Ruggero Loria, Monzer Hamze

**Affiliations:** 1 Laboratoire Microbiologie Santé et Environnement, Doctoral School of Sciences and Technology, Faculty of Public Health, Lebanese University, Tripoli P.O. Box 146404, Lebanon; zahie.abboud1@gmail.com; 2 Istituto Zooprofilattico Sperimentale della Sicilia, Via G. Marinuzzi 3, 90129 Palermo, Italy; galuppolucia@gmail.com (L.G.); maria.vitale@izssicilia.it (M.V.); roberto.puleio@izssicilia.it (R.P.); 3 Dipartimento di Scienze Agrarie, Alimentari e Forestali, University of Palermo, Viale delle Scienze, 90128 Palermo, Italy; marco.tolone@unipa.it; 4 Department of Population Medicine and Diagnostic Sciences, College of Veterinary Medicine, Cornell University, Ithaca, NY 14850, USA

**Keywords:** mastitis, antimicrobial resistance, molecular epidemiology, beta-lactamase, one health, virulence, biofilm, Lebanon

## Abstract

Mastitis is an infectious disease encountered in dairy animals worldwide that is currently a growing concern in Lebanon. This study aimed at investigating the etiology of the main mastitis-causing pathogens in Northern Lebanon, determining their antimicrobial susceptibility profiles, and identifying their antimicrobial resistance (AMR) genes. A total of 101 quarter milk samples were collected from 77 cows and 11 goats presenting symptoms of mastitis on 45 dairy farms. Bacterial identification was carried out through matrix-assisted laser desorption/ionization-time of flight mass spectrometry. Antimicrobial susceptibility was tested by disc diffusion and broth microdilution methods. Molecular characterization included polymerase chain reaction (PCR) screening for genes encoding extended-spectrum beta-lactamases (ESBLs) and plasmid-mediated *AmpC* among *Enterobacterales* isolates, and virulence factors among *Staphylococcus* isolates. *Escherichia coli* isolates were subjected to phylogenetic typing by a quadruplex PCR method. The most frequently identified species were *Streptococcus uberis* (19.2%), *Streptococcus agalactiae* (15.1%), *E. coli* (12.3%), and *Staphylococcus aureus* (10.96%). Gram-positive bacteria were resistant to macrolides and tetracycline, whereas gram-negative bacteria displayed resistance to ampicillin and tetracycline. Two ESBL genes, *bla*_TEM_ (83.3%) and *bla*_OXA_ (16.7%), and one *AmpC* beta-lactamase gene, *bla*_CMY-II_ (16.7%), were detected among six *E. coli* isolates, which mainly belonged to phylogenetic group B1. Among *Staphylococcus* spp., the *mecA* gene was present in three isolates. Furthermore, four isolates contained at least one toxin gene, and all *S. aureus* isolates carried the *ica* operon. These findings revealed the alarming risk of AMR in the Lebanese dairy chain and the importance of monitoring antimicrobial usage.

## 1. Introduction

Mastitis is by far the most widespread and costly disease in dairy cattle worldwide. It has severe and significant economic consequences that include losses in milk production, adverse health effects, early, mandatory culling, costly veterinary services, and additional labor for animal care costs [1,2,3]. It is a well-established disease that affects a high percentage of dairy cows and is of particular concern for farmers in both developed and developing countries [4,5]. Goat farms also suffer economic losses when faced with the burden of mastitis, which is mainly caused by staphylococcal intramammary infections [6,7].

North Lebanon is home to extensive dairy farming and production of milk and its derivatives, which constitute a fundamental part of the Mediterranean diet. Dairy products are consumed daily by different age categories. The dairy sector in Lebanon is also of important economic significance. It represents a leading source of income for producers and families in rural communities, as well as a beneficial opportunity for labor. Approximately 60% of livestock farmers rely on dairy as their main source of income [8]. The development of the dairy sector in Lebanon has led to excessive use of antimicrobials to improve animal health by treating clinical signs of infectious diseases, such as mastitis, and therefore increase their productivity [9]. Several antimicrobial compounds are also currently used as growth promoters in animal production [10].

Mastitis was reported as the most common cause for antimicrobial administration in farming [11]. Because it is so widespread, mastitis is a concerning issue in Lebanon, especially since local farmers and even veterinarians treat infected animals without the necessary support from laboratory services. Conventional antimicrobials used for mastitis therapy are consequently showing reduced effectiveness in dairy cattle. The prolonged misusage of antimicrobial compounds for the treatment of mastitis has contributed to the emergence of antimicrobial resistance (AMR) [12].

Unfortunately, there is neither enough data on the distribution and circulation of pathogens involved in mastitis and their susceptibility patterns to antimicrobials nor on antimicrobial usage in livestock treatment in Lebanon. However, the limited available reports showed high levels of coliform bacteria contamination in traditional Lebanese dairy products and many of these isolates are associated with multi-drug resistance patterns [13,14]. A cross-sectional study also indicated that 27.9% of raw milk samples in Lebanon are contaminated with extended-spectrum beta-lactamase (ESBL)- and/or carbapenemase-producing *Enterobacterales* [14]. Moreover, a high prevalence of ESBL-producing *Escherichia coli* isolates (67.5%) was reported among healthy cattle as well as a remarkable prevalence of ESBL-positive cattle farms (84%) [15]. Similarly, Zouhairi and colleagues stated that 98.7% of *Staphylococcus* isolates from Lebanese dairy-based food products were resistant to at least one antimicrobial drug, with 94% being methicillin-resistant [16]. Indeed, contaminated dairy products may harbor these antimicrobial resistant microorganisms and may pose a threat to public health by acting as vehicles of transmission of these pathogens, which will later be responsible for food-borne diseases. On the other hand, a recent investigation showed the poor knowledge and irresponsibility of the Lebanese population regarding antimicrobial misuse and resistance [17]. What makes the situation more alarming is the wide availability of antimicrobials in the Lebanese market without the requirement of a veterinarian’s prescription [10,18,19].

In this context, the purpose of the current study was to isolate and identify the main pathogenic bacteria that cause clinical mastitis in dairy farms in Northern Lebanon and to determine their susceptibility patterns to the most frequent classes of antimicrobials utilized in veterinary management. This study also investigated the presence of genes related to AMR, and identified the phylogenetic groups of ESBL- and AmpC-positive *Escherichia coli* isolates. We also determined the mosaicism of specific virulence genes among *Staphylococcus* isolates.

## 2. Materials and Methods

### 2.1. Survey Design

A questionnaire-based survey was performed during March and April 2019 on 45 dairy farms located in the North of Lebanon. For each participating dairy farm, a record sheet registering general information about the farm and a short description of the herd (farm name or area/district, species, clinical signs, percentage of suspected animals, therapy history, and laboratory tests) was collected at the time of inclusion.

### 2.2. Sample Collection

**Figure 1:**
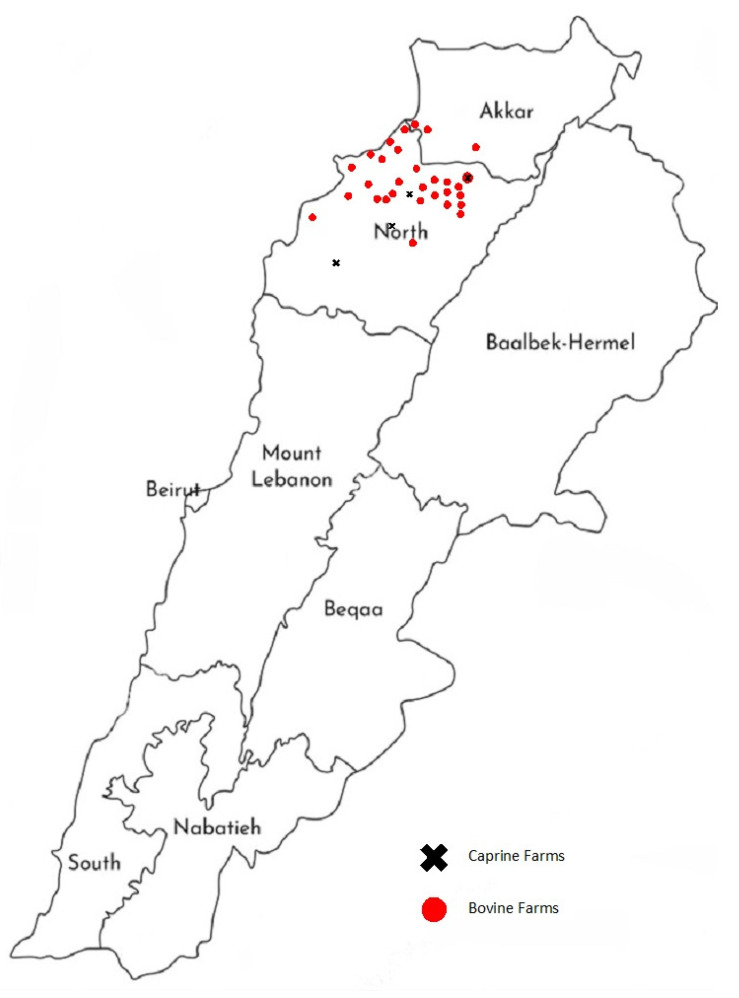
Map of Lebanon displaying the villages in which the dairy farms involved in this study are located.

During March and April 2019, 101 mastitis milk samples (90 cow milk samples and 11 goat milk samples) were aseptically collected from 77 dairy cows and 11 goats showing symptoms of infectious mastitis. Multiple milk samples were at times collected from different quarters of the same cow. Samples were taken from a total of 45 dairy farms located in 32 villages in the Northern region of Lebanon. The villages in which the dairy farms are located are reported in Figure 1. Field veterinarians were responsible for milk sampling. The material supplied for aseptic sampling included disposable gloves and towels, alcohol, cotton, 15 mL sterile sampling tubes, and a specific record sheet for each milk sample. Briefly, teats were cleaned and disinfected using alcohol, the first three streams were discarded, and then milk samples were collected in sterile 15 mL conical tubes. The cooled milk samples were transported on the same day in iceboxes directly to the microbiology laboratory and then processed the same day.

### 2.3. Culturing Techniques and Species Identification

Raw milk samples were examined microbiologically by plating 10 μL of each sample on the following media: Columbia agar with 5% blood; and Columbia CNA Agar with 5% blood, drigalski agar, and mannitol salt agar (all from BioRad, Marnes-la-Coquette, France). The plates were incubated at 37 °C for 24–48 h. Blood agar plates were incubated in 5% CO_2_. The culture was considered positive if there was a growth of individual bacteria in a concentration of more than 10^4^ CFU/mL. All isolates were identified and characterized by matrix-assisted laser desorption/ionization time-of-flight mass spectrometry (MALDI-TOF MS) using a VITEK MS instrument (bioMérieux, Marcy l’Etoile, France).

### 2.4. Antimicrobial Susceptibility Testing

According to the Clinical Laboratory Standard Institute (CLSI) recommendations, bacterial isolates were tested for their antimicrobial susceptibility by standard disk diffusion method either on Mueller–Hinton agar or Mueller–Hinton agar with 5% blood added. The inhibition zones were measured, recorded, and interpreted according to the CLSI guidelines. Minimum inhibitory concentration (MIC) values for all isolates were determined using a broth microdilution method with 96-well microtitre plates (BOPO6, Sensititre, Trek Diagnostic Systems, East Grinstead, UK) containing a total of 18 antimicrobials. The 96-well antimicrobial susceptibility plate contained scaled dilutions for the following 18 antimicrobials: ceftiofur (XNL) (0.25–8), tiamulin (TIA) (0.5–32), chlortetracycline (CTET) (0.5–8), gentamicin (GEN) (1–16), florfenicol (FFN) (0.25–8), oxytetracycline (OXY) (0.5–8), penicillin (PEN) (0.12–8), ampicillin (AMP) (0.25–16), danofloxacin (DANO) (0.12–1), Sulfadimethoxine (SDM) (256), Neomycin (NEO) (4–32), Trimethoprim/sulfamethoxazole (SXT) (2/38), spectinomycin (SPE) (8–64), tylosin tartrate (TYLT) (0.5–32), tulathromycin (TUL) (1–64), tilmicosin (TIL) (4–64), clindamycin (CLI) (0.25–16), and enrofloxacin (ENR) (0.12–2). The plates were incubated aerobically at 37 °C for 24 h (48 h for *Streptococcus* spp.) and were read manually using the Thermo Scientific™ Sensititre™ Manual Viewbox.

### 2.5. Detection of AMR Genes

The primers and their sequences used in the PCRs are listed in Table S1 of the Supplementary Materials. DNA was extracted by a boiling technique. A loop of bacterial colonies was put in a test tube containing 1 mL of sterile 10 mM TRIS 1 mM EDTA (T.E) buffer 0.1% and heated in the Dry Bath (EuroClone, Milan, Italy) at 95 °C for 30 min. The solution was then centrifuged for five minutes at 1000 rpm. The supernatant was collected and used for the downstream process of PCR. Two multiplex PCR reactions were performed to amplify ESBL and *AmpC* beta-lactamase genes in *E. coli* and *Klebsiella oxytoca* isolates, as described by [20]. The first multiplex assay (named Set I) was designed to detect TEM, SHV, CTX-M IV group, and OXA beta-lactamase encoding genes, and the second assay (named Set II) was signed to detect CTX-M I group, CTX-M II group, CMY II, and DHA encoding genes. Additionally, *Staphylococcus* spp. oxacillin-resistant isolates were tested for the presence of the *mecA* and *mecC* genes for confirmation of methicillin resistance.

### 2.6. Determination of Phylogenetic Groups

The distribution of phylogenetic groups amongst ESBL- and/or AmpC-producing *E. coli* isolates was determined as described by Clermont and colleagues [21]. Eight phylogroups are now recognized: seven (A, B1, B2, C, D, E, F) belong to *E. coli* sensu stricto, whereas the eighth is the *Escherichia* cryptic clade I. Phylogenetic groups were identified by multiplex PCR based on the presence or absence of four DNA markers (*chuA*, *yjaA*, DNA fragment *TSPE4.C2*, and *arpA*).

### 2.7. Detection of Biofilm and Virulence Associated Genes of Staphylococcus Spp.

The capability of *Staphylococcus* spp. to form biofilm is considered an additional virulence factor in mastitis cases, due to its ability to adhere to and persist in tissues and the environment [22]. Thus, two separate reactions of PCR were performed to amplify biofilm-associated protein (*bap*) and fragments of the intracellular adhesion (*ica*) genes involved in biofilm formation in *Staphylococcus* spp. as previously described [23]. Moreover, multiplex PCR assays were carried out to amplify staphylococcal enterotoxins (*sea-see, seg, seh, sei, sej, and sep*), exfoliative toxins (*eta* and *etb*), and toxic shock syndrome toxin 1 (*tsst-1*) genes, as recently described in [24].

## 3. Results

### 3.1. Main Findings of the Questionnaire-Based Survey

Our comprehensive questionnaire-based survey revealed that the most prevalent form of mastitis encountered in our study area was clinical mastitis (92%; 81/88), followed by subclinical mastitis (8%; 7/88). Animals had at least one of the following symptoms: abnormal milk appearance (presence of blood in milk and/or watery to viscous milk with clots varying from gray-white to yellowish) (60.2%), hardness and swelling of the udder (47.7%), and/or reduced milk production (45.4%), inappetence (3.4%), diarrhea (2%), chronic inflammation (1.1%), and/or anorexia (1.1%). Empirical antimicrobial treatment was reported in four farms using cefaclor (Cefatek^®^), amoxicillin, tylosin, colistin, gentamicin (10% intramuscular injection), and/or a combination of penicillin and kanamycin (Penikan P^®^). No laboratory tests were performed before and after antimicrobial prescription, suggesting a potential misuse of drugs.

### 3.2. Distribution of Pathogens

In total, sixty-four milk samples (63.4%; 64/101) were positive by conventional culture. Out of these samples, 73 mastitis-causing pathogens were identified, of which 55 were gram-positive bacteria (75.3%; 55/73) and 18 were gram-negative bacteria (24.7%; 18/73). After MALDI-TOF MS analysis, *Streptococcus uberis* was the predominant bacterial species, followed by *Streptococcus agalactiae*, *E. coli*, *Staphylococcus aureus*, and *Trueperella pyogenes* (Table 1).

### 3.3. Antimicrobial Resistance

Selected bacterial species were subjected to AMR studies by disk diffusion and/or MIC methods including *E. coli, K. oxytoca, Pantoea agglomerans, Serratia marcescens, Raoultella ornithinolytica, Pseudomonas aeruginosa, S. aureus, Staphylococcus caprae, Staphylococcus haemolyticus, S. uberis, S. agalactiae*, and *Streptococcus dysgalactiae*. According to the CLSI guidelines, we reported three antimicrobials (oxacillin, erythromycin, and chloramphenicol) tested by the disk diffusion method, and ten commonly used antimicrobials (penicillin, ampicillin, ceftiofur, gentamicin, clindamycin, oxytetracycline, chlortetracycline, florfenicol, enrofloxacin, and trimethoprim/sulfamethoxazole). Of the eight *S. aureus* isolates screened, two (25%) were methicillin-resistant (MRSA). Moreover, the *S. haemolyticus* isolate was methicillin-resistant, but the *S. caprae* isolate was susceptible to methicillin. Regarding *E. coli*, all isolates were resistant to ampicillin, but only 11.1% to ceftiofur. Lower levels of resistance were observed among streptococcal isolates for ampicillin: *S. uberis* (28.6%), *S. agalactiae* (0%), and *S. dysgalactiae* (0%). Nevertheless, *S. agalactiae* showed a high resistance rate against both oxytetracycline and chlortetracycline (100%), followed by *S. uberis* (92.9%) and *S. dysgalactiae* (50%). Similar resistance rates were observed among *E. coli* isolates against chlortetracycline (88.9%) and oxytetracycline (77.8%). However, fortunately, lower resistance rates were reported for florfenicol (55.6%), trimethoprim/sulfamethoxazole (44.4%), enrofloxacin (22.2%), gentamicin (11.1%), and chloramphenicol (0%). For *S. aureus* isolates, low percentages of resistance to both oxytetracycline and chlortetracycline (12.5%), gentamicin (12.5%), and enrofloxacin (0%) were observed. The results of antibiotic resistance of mastitis bacterial pathogens are presented in Table 2. Furthermore, the results obtained showed the presence of seven (77.8%) multidrug-resistant (MDR) *E. coli* out of nine tested isolates, four of which were resistant to three antibiotic classes, and three to five [25]. In addition, one *S. aureus* isolate (12.5%) was MDR.

### 3.4. Molecular Characterization of Clinical Isolates

Out of the *E. coli* isolates, five and one were positive for *bla*_TEM_ and *bla*_CMY-II_ genes, respectively. One *bla*_TEM_ producing *E. coli* carried an additional *bla*_OXA_ gene. However, the *K. oxytoca* isolate did not harbor any ESBL or AmpC beta-lactamase encoding gene. Phylogrouping on beta-lactamase-producing *E. coli* isolates revealed the predominance of the phylogenetic group B1(4/6; 66.7%), followed by B2 and D (1/6; each). On the other hand, methicillin resistance was confirmed by the detection of the *mecA* gene among the three staphylococcal isolates resistant to oxacillin (two *S. aureus* and one *S. haemolyticus*). None of the isolates harbored the *mecC* gene.

This report also investigated the presence of adhesin genes and showed that 40% of staphylococcal isolates were positive for at least one of the classical enterotoxin genes, and two *S. aureus* isolates harbored one or more enterotoxin genes. One *S. aureus* isolate harbored simultaneously the toxic shock syndrome toxin 1 (*tsst-1*) and the *sec* enterotoxin gene. Regarding biofilm formation genes, all the tested isolates were negative for *bap* gene, but all the *S. aureus* isolates were *icaA* positive (Table 3). None of the isolates harbored the exfoliative toxin genes.

## 4. Discussion

AMR is a growing concern in both human and veterinary medicine in Lebanon [10,18,19]. The extensive use of broad-spectrum antimicrobials in farm animals, the food industry, and in human and veterinary medicine, has led to the emergence of MDR organisms in humans, animals, and the environment [9,14,15,18,26,27,28]. The results of the present study revealed that most *E. coli* isolates tested were MDR. Traditional farming and poor hygiene husbandry, typical of Mediterranean countries including Lebanon, enable the quick spread of resistant micro-organisms, which represents a major challenge for developing countries, including Lebanon, as it severely affects the quantity, quality, and safety of animal and food production with significant economic and social consequences [9,10].

Bacterial infections are the predominant cause of bovine and caprine mastitis. In this study, we reported 88 cases of clinical and subclinical mastitis. Only 10% of farms admitted recent administration of antimicrobials. Although 90% of farms did not report the use of antimicrobials, a non-negligible proportion of farmers are not aware of the proper use of antimicrobials or their effects on animals and perform false practices, which are at the root of the spread of AMR [9,10,17]. Several antimicrobials are readily and legally available in the Lebanese market without the requirement of a veterinarian’s prescription [10].

The analysis of bacterial isolates from bulk milk samples showed the predominance of gram-positive bacteria, particularly *S. uberis*, *S. agalactiae*, and *S. aureus*, followed by *E. coli*. The distribution of mastitis bacterial pathogens varies between different geographic areas and even countries. For example, *S. aureus* and coagulase-negative staphylococci (CNS) have been reported as the most common bovine mastitis in the Middle East, North Africa, and Europe [29,30,31,32,33,34,35,36,37]. In the same context, a large-scale epidemiological study conducted in Italy reported the predominance of *S. agalactiae* and *S. aureus* in dairy herds [38]. Moreover, studies performed in European Mediterranean countries highlighted a significant presence of *E. coli* and *Staphylococcus* spp. [39,40,41,42]. Moreover, a high incidence of *E. coli* was also observed among bovine mastitis in India and Taiwan [43,44]. In addition, one mastitis case of *Streptococcus pneumoniae* was documented. *Pneumococcus* is a commensal bacterium that normally colonizes the human nasopharyngeal cavity and is transmitted by droplets and aerosols either from infected patients or healthy carriers [45]. Although this species is rarely reported in mastitis, various epidemiological studies showed its high prevalence in the Lebanese community [46,47]. This allows us to speculate that there is a potential threat of contamination by handlers. Antimicrobial susceptibility testing showed a high percentage of resistance among most gram-positive and gram-negative isolates against both oxytetracycline and chlortetracycline. This is in line with a previous report which described that 98.4% of streptococcal isolates from dairy cows with mastitis in China were resistant to tetracycline [48]. In Lebanon, a similar percentage of resistance was observed. Most *E. coli* (82.5%) isolated from healthy adult cattle showed tetracycline resistance [15]. Numerous veterinary infectious diseases among cattle are the most frequently treated with oxytetracyclines, explaining the high resistance rates observed in this study [49].

In contrast to streptococcal isolates, which showed very low susceptibility results to erythromycin and clindamycin, none of the staphylococcal isolates showed resistance to these antimicrobials. Although no similar previous study was found in Lebanon, our findings are consistent with similar data reported across the world [42,50,51]. In the human field in Lebanon, a recent cross-sectional study displayed the absence of macrolide and lincosamide resistance in both MRSA and methicillin-sensitive *S. aureus* (MSSA) isolates among food handlers [52]. However, previous Lebanese studies on group A streptococci human isolates showed lower resistance to erythromycin and clindamycin [53,54].

MRSA was isolated twice (25%) in this study. This percentage is consistent with clinical human studies at the nationwide level showing a similar prevalence of this resistance pattern. A compilation of antimicrobial susceptibility data of *S. aureus* from a network of 13 Lebanese hospitals indicated that 28% were MRSA [55]. Moreover, a previous study conducted in the same geographic area reported that 23.8% of *S. aureus* isolates were MRSA [52]. However, MRSA prevalence in the present study is significantly higher than that of other countries such as Croatia [56] and Ukraine [57].

Regarding *E. coli*, 100% and 11.1% of isolates were resistant to ampicillin and ceftiofur, respectively. Despite this, only one isolate presented resistance to ceftiofur using the phenotypic method. Third-generation cephalosporins resistance was screened by two multiplex PCR reactions as described by [20]. Overall, 55.5% of *E. coli* isolates were positive for ESBL and plasmid-mediated AmpC beta-lactamase genes: *bla*_TEM_, *bla*_CMY-II_, and/or *bla*_OXA_. Unfortunately, due to logistical reasons, this study did not evaluate the susceptibility of isolates to other third-generation cephalosporins such as cefotaxime, ceftriaxone, ceftazidime, and cefixime. This study confirmed that food animals and foodstuffs are a well-known reservoir of ESBL-producing *E. coli* in Lebanon. Wide dissemination of third-generation cephalosporin resistance, coupled with resistances to carbapenems, colistin, and numerous other antimicrobial compounds, has been reported among animals in the last decade [9,14,15,18,58]. The phylogenetic typing analysis carried out on the ESBL-producing *E. coli* isolates revealed that most of them belong to phylogroups B1 (66.7%) followed by both B2 and D (16.7% each), which have previously been reported in clinical third-generation cephalosporin-resistant isolates in Lebanon [19]. Phylogenetic grouping is an important approach to understanding the pathogenicity and evolutionary relationships between different strains [59]. No association was identified between AMR and phylogroup due to the limited number of *E. coli* isolates. Thus, a larger study on a higher number of isolates is needed. At the worldwide level, the majority of *E. coli* mastitis strains belong to phylogenetic groups A and B1 [60]. A high frequency of phylogroup B1 was also observed in an Iranian study evaluating the phylogeny of *E. coli* isolated from clinical mastitis [61]. A Brazilian study revealed that most *E. coli* isolates from bovine mastitis belonged to phylogenetic group A (52%), followed by B1 (38%) [62]. Similarly, other studies from Ireland, Switzerland, Serbia, and China have noted that *E. coli* isolates were mainly assigned to the phylogenetic groups A and B1 [63,64,65,66,67].

The ability of *S. aureus* isolates to form biofilms is an important mechanism that reinforces pathogenicity and contributes to AMR. PCR results showed that all *S. aureus* isolates were positive for the intracellular adhesion gene *icaA,* while the *bap* gene was not identified in any of the isolates. Our findings follow previous studies conducted in Poland [68], New Zealand [22], and the United Kingdom [69] that detected the unique presence of the *icaA* gene among all *S. aureus* isolates from bovine mastitis. Our study also showed that 40% and 25% of staphylococcal and *S. aureus* isolates were positive for at least one enterotoxin gene, respectively. Interestingly, one *S. aureus* isolate simultaneously harbored *tsst-1* and *sec* genes, predicting the presence of the staphylococcal pathogenicity island 1 (SaPI1), as described previously [70].

## 5. Conclusions

This study provides a consistent source of relevant data on AMR levels and trends of mastitis bacterial pathogens in Lebanese dairy bovines and caprines. Despite resistance rate variations, our findings confirmed the wide dissemination of antimicrobial-resistant bacteria in the Lebanese dairy industry. Many causative agents isolated in this study are zoonotic and can be transmitted directly between animals and humans or through the food chain. Therefore, there is a drastic need for a national strategy based on the one health approach to address the AMR issue and regulate the usage of antimicrobials in the veterinary sector in Lebanon, including bans on over-the-counter drugs and growth promoters. Additionally, the training and education of farmers is necessary to create awareness of AMR through effective communication, education, and training. Further large-scale, nationwide studies are also needed to evaluate the correlations between the use of antimicrobials in common husbandry practices and the onset of AMR in Lebanon.

## Figures and Tables

**Table 1:** Bacterial isolates from clinical and subclinical bovine and caprine mastitis cases.

Bacterial Species	Animal Origin		Status of Infection		Percentage (%)
	Cow	Goat	Clinical	Subclinical	
*Streptococcus uberis*	14		14		19.2
*Streptococcus agalactiae*	11		11		15.1
*Escherichia coli*	7	2	9		12.3
*Staphylococcus aureus*	7	1	8		11
*Trueperella pyogenes*	5	2	7		9.6
*Aerococcus viridans*	4		4		5.5
*Raoultella ornithinolytica*	3		3		4.1
*Streptococcus dysgalactiae*	2		2		2.7
*Streptococcus pluranimalium*	1	1	1	1	2.7
*Corynebacterium bovis*	2			2	2.7
*Corynebacterium xerosis*	2			2	2.7
*Pseudomonas aeruginosa*	2		2		2.7
*Staphylococcus caprae*		1		1	1.4
*Staphylococcus haemolyticus*		1		1	1.4
*Pantoea agglomerans*	1		1		1.4
*Pasteurella multocida*	1		1		1.4
*Klebsiella oxytoca*	1		1		1.4
*Serratia marcescens*	1		1		1.4
*Streptococcus pneumoniae*	1		1		1.4
Total	65	8	66	7	100%

**Table 2:** Percentage of resistant *Enterobacterales*, *Pseudomonas* spp., *Staphylococcus* spp., and *Streptococcus* spp. isolates recovered from milk samples with bovine and caprine mastitis cases.

	N	PEN ^‡^	AMP ^‡^	XNL ^‡^	GEN ^‡^	ERY ^‼^	CLI ^‡^	OXY ^‡^	CTET ^‡^	CHL ^‼^	FFN ^‡^	ENR ^‡^	SXT ^‡^
*Escherichia coli*	9	-	100	11.1	11.1	-	-	77.8	88.9	0	55.6	22.2	44.4
*Klebsiella oxytoca*	1	-	100	0	0	-	-	100	100	0	0	0	0
*Pantoea agglomerans*	1	-	0	0	0	-	-	0	100	0	0	100	0
*Serratia marcescens*	1	-	100	0	0	-	-	100	100	0	100	0	0
*Raoultella ornithinolytica*	3	-	100	0	0	-	-	0	100	0	0	0	0
*Pseudomonas aeruginosa*	2	-	-	-	0	-	-	-	-	-	-	50	-
*Staphylococcus aureus*	8	62.5	50	-	12.5	0	0	12.5	12.5	0	-	0	0
*Staphylococcus caprae*	1	100	100	-	0	0	0	0	100	0	-	0	0
*Staphylococcus haemolyticus*	1	100	100	-	0	0	0	100	100	0	-	0	0
*Streptococcus uberis*	14	14.3	28.6	14.3	-	71.4	100	92.9	92.9	42.9	-	50	0
*Streptococcus agalactiae*	10	0	0	0	-	81.8	100	100	100	18.2	-	100	0
*Streptococcus* *dysgalactiae*	2	0	0	0	-	50	50	50	50	50	-	0	0

^‡^ Determined using the broth microdilution method; ^‼^ Determined using the disk diffusion method. PEN—penicillin; OXA—oxacillin; AMP—ampicillin; XNL—ceftiofur; GEN—gentamicin; ERY—erythromycin; CLI—clindamycin; OXY—oxytetracycline; CTET—chlortetracycline; CHL—chloramphenicol; FFN—florfenicol; ENR—enrofloxacin; SXT—trimethoprim/sulfamethoxazole.

**Table 3:** Distribution of genes among *Staphylococcus* spp. isolates.

Isolate	Enterotoxins	Exfoliative Toxins and Toxic Shock Syndrome Toxin-1	Methicillin Resistance	Biofilm Formation
*S. aureus* AL 081			*mecA*	*ica operon*
*S. aureus* AL 084	*sei, seg*		*mecA*	*ica operon*
*S. aureus* AL 085				*ica operon*
*S. aureus* AL 086				*ica operon*
*S. aureus* AL 087				*ica operon*
*S. aureus* AL 088				*ica operon*
*S. aureus* AL 089	*sec*	*tsst-1*		*ica operon*
*S. aureus* AL 090				*ica operon*
*S. haemolyticus* AL 091	*sea, sej, sep*		*mecA*	
*S. caprae* AL 092	*seb*			

## Supplementary Materials

The following are available online at https://www.mdpi.com/article/10.3390/microorganisms9061148/s1, Table S1—Primer sequences used in PCRs.
