# Supplementary material for: Molecular Characterization of Antimicrobial Resistance and Virulence Genes of Bacterial Pathogens from Bovine and Caprine Mastitis in Northern Lebanon
Source: Microorganisms. 2021 May 27;9(6):1148. doi: 10.3390/microorganisms9061148 (PMC8228836; doi:10.3390/microorganisms9061148)
Supplement: Supplementary file 1 [file microorganisms-09-01148-s001.zip › microorganisms-1222938-supplementary.pdf]

**Table S1.** Primer sequences used in PCRs					
Gene	Primer name	Strand	Nucleotide Sequence (5′-3′)	Fragment size (bp)	Reference
ica	icaH-7c	Forward	CTTTCGTTATAACAGGCAAG	~700	Cucarella et al., 2004 [1]
	icaH-1m	Reverse	TATACCTTTCTTCGATGTCG		
bap	sasp-7c	Forward	GCTGTTGAAGTTAATACTGTACCTGC	~1000	Cucarella et al., 2004 [1]
	sasp-6m	Reverse	CCCTATATCGAAGGTGTAGAATTGCAC		
$bla_{CTX-M-4}$	CTX-M IV	Forward	GACAAAGAGAGTGCAACGGATG	501	Kim et al., 2009 [2]
		Reverse	TCAGTGCGATCCAGACGAAA		
bla_{TEM}	TEM	Forward	AGTGCTGCCATAACCATGAGTG	431	Kim et al., 2009 [2]
		Reverse	CTGACTCCCC GTCGTGTAGATA		
bla_{OXA}	OXA	Forward	ATTATCTACAGCAGCGCCAGTG	296	Kim et al., 2009 [2]
		Reverse	TGCATCCACGTCTTTGGTG		
bla_{SHV}	SHV	Forward	GATGAACGCTTTCCCATGATG	214	Kim et al., 2009 [2]
		Reverse	CGCTGTTATCGCTCATGGTAA		
bla_{CMY-2}	CMY II	Forward	AGCGATCCGGTCACGAAATA	695	Kim et al., 2009 [2]
		Reverse	CCCGTTTTATG CACCCATGA		
$bla_{CTX-M-1}$	CTX-M I	Forward	TCCAGAATAAGGAATCCCATGG	621	Kim et al., 2009 [2]
		Reverse	TGCTTTACCCAGCGTCAGAT		
$bla_{CTX-M-2}$	CTX-M II	Forward	ACCGCCGATAATTCGCAGAT	588	Kim et al., 2009 [2]
		Reverse	GATATCGTTGGTGGTGCCATAA		
bla_{DHA}	DHA	Forward	GTGGTGGACAGCACCATTAAA	314	Kim et al., 2009 [2]
		Reverse	CCTGCGGTATAGGTAGCCAGAT		
chuA	chuA.1b	Forward	ATGGTACCGGACGAACCAAC	288	Clermont et al., 2013 [3]
	chuA.2	Reverse	TGCCGCCAGTACCAAAGACA		Clermont et al., 2000 [4]
yjaA	yjaA.1b	Forward	CAAACGTGAAGTGTCAGGAG	211	Clermont et al., 2013 [3]
	yjaA.2b	Reverse	AATGCGTTCCTCAACCTGTG		Clermont et al., 2013 [3]
TspE4C2	TspE4C2.1b	Forward	CACTATTCGTAAGGTCATCC	152	Clermont et al., 2013 [3]
	TspE4C2.2b	Reverse	AGTTTATCGCTGCGGGTCGC		Clermont et al., 2013 [3]
arpA	AceK.f	Forward	AACGCTATTCGCCAGCTTGC	400	Clermont et al., 2013 [3]

	ArpA1.r	Reverse	TCTCCCCATACCGTACGCTA		Clermont et al., 2004 [5]
arpA	ArpAgpE.f	Forward	GATTCCATCTTGTCAAAATATGCC	301	Lescat et al., 2013 [6]
	ArpAgpE.r	Reverse	GAAAAGAAAAAGAATTCCCAAGAG		
trpA	trpAgpC.1	Forward	AGTTTTATGCCCAGTGCGAG	219	Lescat et al., 2013 [6]
	trpAgpC.2	Reverse	TCTGCGCCGGTCACGCCC		
sea	GSEAR-1	Forward	GGTTATCAATGTGCGGGTGG	102	Mehrota et al., 2000 [7]
	GSEAR-2	Reverse	CGGCACTTTTTTCTCTTCGG		
seb	GSEBR-1	Forward	GTATGGTGGTGTAACTGAGC	164	Mehrota et al., 2000 [7]
	GSEBR-2	Reverse	CCAAATAGTGACGAGTTAGG		
sec	GSECR-1	Forward	AGATGAAGTAGTTGATGTGTATGG	451	Mehrota et al., 2000 [7]
	GSECR-2	Reverse	CACACTTTTAGAATCAACCG		
sed	GSEDR-1	Forward	CCAATAATAGGAGAAAATAAAAG	278	Mehrota et al., 2000 [7]
	GSEDR-2	Reverse	ATTGGTATTTTTTTTCGTTC		
see	GSEER-1	Forward	AGGTTTTTTCACAGGTCATCC	209	Mehrota et al., 2000 [7]
	GSEER-2	Reverse	CTTTTTTTTCTTCGGTCAATC		
seg	SEG-F	Forward	GTTAGAGGAGGTTTTATG	198	Bania et al., 2006 [8]
	SEG-R	Reverse	TTCCTTCAACAGGTGGAGA		
seh	SEH-F	Forward	CAACTGCTGATTTAGCTCAG	173	Bania et al., 2006 [8]
	SEH-R	Reverse	CCCAAACATTAGCACCA		
sei	SEI-F	Forward	GGCCACTTTATCAGGACA	328	Bania et al., 2006 [8]
	SEI-R	Reverse	AACTTACAGGCAGTCCA		
sej	SEJ-F	Forward	GTTCTGGTGGTAAACCA	131	Bania et al., 2006 [8]
	SEJ-R	Reverse	GCGGAACAACAGTTCTGA		
sep	SEP-F	Forward	TCAAAAGACACCGCCAA	396	Bania et al., 2006 [8]
	SEP-R	Reverse	ATTGTCCTTGAGCACCA		
*fem*A	GFEMAR-1	Forward	AAAAAAGCACATAACAAGCG	132	Mehrota et al., 2000 [7]
	GFEMAR-2	Reverse	GATAAAGAAGAAACCAGCAG		
*mec*A	GMECAR-1	Forward	ACTGCTATCCACCCTCAAAC	163	Mehrota et al., 2000 [7]
	GMECAR-2	Reverse	CTGGTGAAGTTGTAATCTGG		
eta	GETAR-1	Forward	GCAGGTGTTGATTTAGCATT	93	Mehrota et al., 2000 [7]
	GETAR-2	Reverse	AGATGTCCCTATTTTTGCTG		
etb	GETBR-1	Forward	ACAAGCAAAAGAATACAGCG	226	Mehrota et al., 2000 [7]
	GETBR-2	Reverse	GTTTTTGGCTGCTTCTCTTG		
tst	GTSSTR-1	Forward	ACCCCTGTTCCCTTATCATC	326	Mehrota et al., 2000 [7]
	GTSSTR-2	Reverse	TTTTCAGTATTTGTAACGCC		

References

1. Cucarella, C.; Tormo, M.A.; Ubeda, C.; Trotonda, M.P.; Monzón, M.; Peris, C.; Amorena, B.; Lasa, I.; Penadés, J.R. Role of biofilm-associated protein bap in the pathogenesis of bovine *Staphylococcus aureus*. *Infect Immun* **2004**, *72*, 2177-2185, doi:10.1128/iai.72.4.2177-2185.2004.
2. Kim, J.; Jeon, S.; Rhie, H.; Lee, B.; Park, M.; Lee, H.; Lee, J.; Kim, S. Rapid detection of extended spectrum β-Lactamase (ESBL) for *Enterobacteriaceae* by use of a multiplex PCR-based method. *Infect Chemother* **2009**, *41*, 181-184.
3. Clermont, O.; Christenson, J.K.; Denamur, E.; Gordon, D.M. The Clermont *Escherichia coli* phylo-typing method revisited: improvement of specificity and detection of new phylo-groups. *Environ Microbiol Rep* **2013**, *5*, 58-65, doi:10.1111/1758-2229.12019.
4. Clermont, O.; Bonacorsi, S.; Bingen, E. Rapid and simple determination of the *Escherichia coli* phylogenetic group. *Appl. Environ. Microbiol* **2000**, *66*, 4555-4558, doi:10.1128/aem.66.10.4555-4558.2000.
5. Clermont, O.; Bonacorsi, S.; Bingen, E. characterization of an anonymous molecular Marker strongly linked to *Escherichia coli* strains causing neonatal meningitis. *J Clin Microbiol* **2004**, *42*, 1770-1772, doi:10.1128/jcm.42.4.1770-1772.2004.
6. Lescat, M.; Clermont, O.; Woerther, P.L.; Glodt, J.; Dion, S.; Skurnik, D.; Djossou, F.; Dupont, C.; Perroz, G.; Picard, B.; et al. Commensal *Escherichia coli* strains in Guiana reveal a high genetic diversity with host-dependant population structure. *Environ Microbiol Rep* **2013**, *5*, 49-57, doi:10.1111/j.1758-2229.2012.00374.x.
7. Mehrotra, M.; Wang, G.; Johnson, W.M. Multiplex PCR for detection of genes for *Staphylococcus aureus* enterotoxins, exfoliative toxins, toxic shock syndrome toxin 1, and methicillin resistance. *J Clin Microbiol* **2000**, *38*, 1032-1035.
8. Bania, J.; Dabrowska, A.; Bystron, J.; Korzekwa, K.; Chrzanowska, J.; Molenda, J. Distribution of newly described enterotoxin-like genes in *Staphylococcus aureus* from food. *Int J Food Microbiol* **2006**, *108*, 36-41, doi:10.1016/j.ijfoodmicro.2005.10.013.
